# PKA regulatory subunit R2B is required for murine and human adipocyte differentiation

**DOI:** 10.1530/EC-13-0049

**Published:** 2013-11-18

**Authors:** Erika Peverelli, Federica Ermetici, Sabrina Corbetta, Ettore Gozzini, Laura Avagliano, Marco A Zappa, Gaetano Bulfamante, Paolo Beck-Peccoz, Anna Spada, Giovanna Mantovani

**Affiliations:** 1 Endocrinology and Diabetology Unit, Department of Clinical Sciences and Community HealthUniversity of Milan, Fondazione IRCCS Ca' Granda Ospedale Maggiore PoliclinicoPad.Granelli Via F. Sforza 3520122, MilanItaly; 2 Diabetology and Metabolic Disease UnitIRCCS Policlinico San DonatoSan Donato MilaneseItaly; 3 Endocrinology Unit, IRCCS Policlinico S.Donato, Department of Biomedical Sciences for HealthUniversity of MilanMilanItaly; 4 Department of Health Sciences, Unit of Human Pathology – San Paolo Hospital Medical SchoolUniversity of MilanMilanoItaly; 5 Surgical DepartmentOspedale Sacra Famiglia FatebenefratelliErbaItaly

**Keywords:** PKA, cAMP, differentiation, adipocytes

## Abstract

Adipogenesis is a complex process modulated by several factors, including cAMP signaling. The main cAMP target is protein kinase A (PKA), a tetrameric enzyme with four regulatory subunits showing tissue-specific expression and function: PRKAR2B is the main regulatory subunit in adipose tissue in mice and in adult humans. This study aimed to evaluate the expression of PKA regulatory subunits in human adipose tissue during fetal development and to investigate their role in the differentiation of 3T3-L1 and primary human preadipocytes. The expression of PKA regulatory subunits was evaluated in fetal adipose tissue (immunohistochemistry) and in cultured 3T3-L1 and primary human preadipocytes (western blot analysis). Cultured cells were transiently transfected with siRNA against *PRKAR2B* and induced to differentiate. Differentiation was evaluated by intracellular triglyceride staining (Oil Red O) and expression of molecular markers of adipocyte differentiation. In this study, we found that PRKAR2B is the main regulatory subunit in human adipose tissue during fetal development, from 12 weeks of gestation to the end of gestation, as well as in 3T3-L1 and primary human preadipocytes. The expression of PRKAR2B increases progressively during *in vitro* differentiation. The silencing of *PRKAR2B* abolishes the increase in the expression of peroxisome proliferator-activated receptor gamma (PPARγ (PPARG)), fatty acid synthase, aP2 (FABP4), and lipoprotein lipase, as well as intracellular triglyceride accumulation, resulting in impaired adipocyte differentiation in both mouse and human cell systems. In conclusion, PRKAR2B is the key PKA regulatory subunit involved in mouse and human adipose tissue development. The physiological increase in the expression of PRKAR2B is an essential event in adipogenesis in both mice and humans, and it might represent a possible target for future strategies for obesity treatment.

## Introduction

White adipose tissue (WAT) is mainly involved in energy storage and mobilization in the form of triglycerides, although a paracrine and endocrine function of white adipocytes has also been recognized. Excess of WAT results from an increase in both adipocyte size and number, and it is associated with obesity and related metabolic disorders. Adipogenesis is a complex, not completely clarified process characterized by a cascade of gene activation processes tightly controlled by a myriad of different factors (1, 2), which have been only partially elucidated.

cAMP acts as an intracellular second messenger in the regulation of a number of different cellular processes, such as cell growth and differentiation. It is known that cAMP signaling also affects adipogenesis, through different and partially unexplored mechanisms. Indeed, 3-isobutyl-1-methylxanthine (IBMX), one of the inducers of adipogenesis, acts through the increase in intracellular cAMP levels. The activation of cAMP-responsive element-binding protein (CREB) induces the expression of several transcription factors that promote adipogenesis, mainly CCAAT/enhancer-binding proteins (*C/EBP*s) and peroxisome proliferator-activated receptor gamma (*PPARγ* (*PPARG*)) (3, 4, 5, 6). In addition, the increase in intracellular cAMP levels leads to the production of a putative endogenous PPARγ ligand (7). Although a role for exchange protein directly activated by cAMP (EPAC (RAPGEF3)) in cAMP-mediated adipocyte differentiation has been suggested (8), evidence indicates that the activation of CREB is mainly modulated by cAMP-dependent protein kinase A (PKA) (9).

PKA is a tetrameric enzyme composed of two catalytic and two regulatory subunits. There are four regulatory subunits (R1A, R1B, R2A, and R2B) differently expressed in mammalian tissues, affecting cAMP-dependent functions (10). In adipose tissue, the major holoenzyme assembled under normal conditions contains the R2B regulatory subunit, even though both PRKAR2B and PRKAR1A are expressed in fat cells (11, 12). Targeted disruption of the *Prkar2b* gene in mice leads to stable alterations in energy storage and utilization, resulting in a lean phenotype (13). It has been reported that human adipose tissue exhibits important BMI-related differences in the expression and activity of PRKAR2B that might contribute to the variability of cAMP-stimulated lipolysis in obese and nonobese subjects (12). However, data on the expression and function of the different PKA regulatory subunits during adipose tissue differentiation are few and contradictory (14, 15). Moreover, these studies have been carried out in murine cell line models, mainly 3T3-L1 preadipocytes, while studies in human models of adipocyte differentiation are still scarce.

In this study, we evaluated the expression of PKA regulatory subunits in human adipose tissue during fetal development as well as their role in the differentiation process of two *in vitro* cell models of adipogenesis, i.e., 3T3-L1 and primary human preadipocytes. Our data showed that the physiological increase in the expression of PRKAR2B is an essential event in adipogenesis in both mice and humans.

## Materials and methods

### Fetal tissues

Tissues from fetuses of gestational ages 12, 14, 20, 25, and 36 weeks were collected, as described previously (16). Briefly, fetuses of gestational ages 12, 14, 20, and 25 weeks were collected after legal voluntary termination of pregnancy for maternal psychiatric disorders, according to the Italian legislation that allows termination of pregnancy for medical reasons up to 25 weeks of gestation. Fetal death at 36 weeks was due to abruptio placentae for unexplained reasons. In all cases, informed consent of the mother was obtained before procurement of the tissues, in accordance with the guidelines outlined by the San Paolo Institute Ethics Committee. Samples were fixed in 10% buffered formalin and paraffin-embedded for immunohistochemistry (IHC). At least two samples of each fetal tissue from two different fetuses at each given trimester of gestation were analyzed in three separate experiments.

### Immunohistochemistry

Sections from paraffin-embedded fetal tissues were processed for IHC, as reported previously (17). A polyclonal antibody against human delta-like protein 1 (DLK1), also known as preadipocyte factor 1 (Pref1) (Abcam, Cambridge, UK), was used to identify fibroblast-like preadipocytes not identifiable by morphological evaluation. Specific MABs against PRKAR1A, PRKAR2A, and PRKAR2B were used under the conditions specified by the manufacturer (BD Transduction Laboratories, Lexington, UK). Antigen–antibody detection was carried out using the DAKO ChemMate En Vision detection kit (DAKO A/S, Glostrup, Denmark) according to the manufacturer's instructions. Sections were stained with 3,3′-diaminobenzidine substrate and counterstained with Mayer's hematoxylin, and slides were prepared for light microscopy examination, as described previously (17). Negative controls were obtained by occulting the primary antibody or by using an unrelated mouse MAB.

### Cell preparation, culture, and differentiation

Murine 3T3-L1 preadipocytes (kindly provided by J A M Maier, Milan, Italy) were grown until confluence at 37 °C and in 5% CO_2_ in DMEM supplemented with 10% calf serum and 100 units/ml penicillin and 100 μg/ml streptomycin. Two days after confluence, differentiation was induced (day 0) by changing the growth medium to differentiation medium, containing DMEM with 10% fetal bovine serum (FBS), 0.5 mmol/l IBMX, 1 μmol/l dexamethasone, and 10 μg/ml insulin; after 48 h, the cells were maintained in DMEM with 10% FBS and the medium was changed every 2 days. The cells differentiated within 8 days.

Human subcutaneous adipose tissue samples from normal (BMI between 18.5 and 25 kg/m^2^) healthy subjects were collected from the surgical incision site during abdominal surgical procedures for benign disease (elective cholecystectomy). The study was approved by the local ethics committee, and all the subjects gave informed consent before participation, according to the Declaration of Helsinki. The samples were digested with collagenase type 2 (Sigma–Aldrich), and preadipocytes were isolated as described previously (12), on the basis of cell dimension and density.

Primary human preadipocytes were cultured until they reached the total confluence stage in complete Preadipocyte Growth Medium (PromoCell GmbH, Heidelberg, Germany), a low-serum medium optimized for primary human cells containing DMEM F12 with 5% fetal calf serum (FCS), 0.4% endothelial cell growth supplement, 10 ng/ml epidermal growth factor (recombinant human), and 1 μg/ml hydrocortisone. After confluence, the growth medium was replaced with Preadipocyte Differentiation Medium (Promocell GmbH), a serum-free medium containing DMEM F12 with 8 μg/ml d-biotin, 0.5 μg/ml insulin (recombinant human), 400 ng/ml dexamethasone, 44 μg/ml IBMX, 9 ng/ml l-thyroxine, and 3 μg/ml ciglitazone. The cells were maintained in Preadipocyte Differentiation Medium for 72 h to induce the differentiation of preadipocytes into mature adipocytes. Then, the medium was changed to Adipocyte Nutrition Medium (Promocell GmbH), containing DMEM F12 with 3% FCS, 8 μg/ml d-biotin, 0.5 μg/ml insulin (recombinant human), and 400 ng/ml dexamethasone. The cells were fed every 2–3 days with fresh Adipocyte Nutrition Medium to complete the differentiation process. The cells differentiated after 12–14 days.

### Oil Red O staining

The cells were washed three times with PBS, fixed in 10% formalin in PBS for 10 min at room temperature, washed once again with PBS, and then stained with 60% filtered Oil Red O stock solution (0.35 g Oil Red O (Sigma–Aldrich) in 100 ml isopropanol) for 20 min, washed four times with water, and then analyzed under a microscope. The cells were photographed using a phase-contrast microscope (Nikon Eclipse Ti inverted microscope; objective: Nikon CFI Plan Achromat DL 20×).

### Synthesis and transfection of siRNA

siRNAs against mouse and human *PRKAR2B* genes (*R2B* siRNA) were obtained from Dharmacon (Chicago, IL, USA).

Subconfluent 3T3-L1 cells were transfected with double-stranded RNA using the siPORT NeoFX transfection reagent (Ambion, Austin, TX, USA), according to the manufacturer's instructions. Both siRNA (10 nM) and siPORT NeoFX were diluted in Opti-MEM I Reduced-Serum Medium (Invitrogen). The cells were exposed to double-stranded RNA and transfection reagent for 24 h. Then, the culture medium was replaced with fresh normal growth medium.

Primary human preadipocytes were transfected with double-stranded RNA using the HiPerFect Transfection Reagent (Qiagen), according to the manufacturer's instructions. Similar to the procedure followed for 3T3-L1 cells, siRNA was diluted in Opti-MEM I Reduced-Serum Medium. The cells were exposed to double-stranded RNA (10 nM) and transfection reagent for 72 h. Then, the culture medium was replaced with fresh normal growth medium.

In order to identify nonspecific effects, a negative control siRNA, a nontargeting sequence that has no significant homology to the sequence of human, mouse, or rat transcripts, was used in each experiment. siRNA against *GAPDH* was used as an internal positive control. Western blot analysis was carried out in each experiment to determine the expression levels of PRKAR2B in the silenced cells.

Preliminary experiments to determine the optimal concentration of siRNAs and the kinetics of *PRKAR2B* silencing were carried out. The efficiency of silencing was about 60% in both the cell models. Silencing was effective after 48 and 72 h in 3T3-L1 and primary human preadipocytes respectively, and it lasted until 10–11 days in both the cell models.

### Western blot analysis and mitotic clonal expansion evaluation

Western blot analysis was carried out, as described previously (17), on total protein extracts from 3T3-L1 cells and primary human preadipocytes. Antibodies against PRKAR1A, PRKAR2A, PRKAR2B, and catalytic subunits were obtained from BD (BD Transduction Laboratories). Polyclonal antibodies against both PPARγ 1–2 and lipoprotein lipase (LPL) were obtained from Santa Cruz Biotechnology. Antibodies against aP2 (fatty acid-binding protein 4, FABP4) and fatty acid synthase (FAS) were obtained from GeneTex (Irvine, CA, USA). MAB against GAPDH was obtained from Ambion.

Chemiluminescence was detected using the ChemiDoc-IT Imaging System (UVP, Upland, CA, USA) and analyzed using the image analysis program NIH ImageJ.

All the experiments were repeated at least three times with comparable results. The experiments on primary cell cultures were carried out on at least three different samples from different subjects.

To determine mitotic clonal expansion (MCE), 3T3-L1 cells were transfected with *R2B* or negative control siRNA for 72 h and then induced or not induced to differentiate. After 48 h, the cells were trypsinized, and the number of cells was determined using a hemocytometer.

### Statistical analysis

The results are expressed as means±s.d. The unpaired two-tailed Student's *t*-test was used to determine the significance of differences between two series of data. *P*<0.05 was classified as statistically significant.

## Results

### PRKAR2B expression in human adipose tissue during fetal development

In order to investigate the expression of PKA regulatory subunits in human adipose tissue during fetal development, IHC was carried out on tissue sections of adipose tissue obtained from fetuses of different gestational ages. To identify preadipocytes within the mesenchymal tissue surrounding organs in fetuses of early gestational ages, IHC with an antibody against Pref1, a marker of early preadipocytes, was carried out.

IHC indicated that PRKAR2B was the main regulatory subunit in human adipose tissue during fetal development, starting from 12 weeks of gestation. In particular, IHC revealed a weak immunopositivity for PRKAR2B, which co-localized with Pref1, in fibroblast-like preadipocytes in periorbital and retroperitoneal mesenchymal tissue obtained from fetuses of gestational ages 12 and 14 weeks and the absence of PRKAR1A.

**Figure 1:**
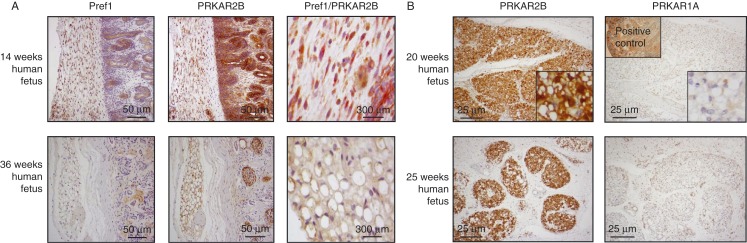
(A) Immunohistochemistry for Pref1 and PRKAR2B carried out in human perirenal adipose tissue of 14-week and 36-week-old fetuses, as representative examples. Immunostaining for Pref1 was carried out to identify fibroblast-like preadipocytes. Positive immunostaining for Pref1 was observed in the adipose tissue of a 14-week-old fetus, positive immunostaining was also observed for PRKAR2B. Double immunostaining for Pref1 (red) and PRKAR2B (brown) was observed in the preadipocytes of the 14-week-old fetus. The adipose tissue of the 36-week-old fetus was mainly composed of mature adipocytes (Pref1−). Positive immunoreactivity for PRKAR2B was observed in mature adipocytes of the 36-week-old fetus. Magnification ×20. Panels on the right, magnification ×120. (B) Immunostaining for PRKAR2B and PRKAR1A carried out in human retroperitoneal adipose tissue of 20-week-old (adrenal) and 25-week-old (bladder) fetuses, as representative examples. Visceral adipose tissue from fetuses of gestational ages 20 and 25 weeks was composed of both preadipocytes and mature adipocytes (see inset), with intense immunopositivity for PRKAR2B and low immunopositivity for PRKAR1A protein. Magnification ×10, inset ×40. Intense immunopositivity for PRKAR1A in human kidney sample is shown as a positive control. Magnification ×10.

Visceral adipose tissue almost entirely composed of mature adipocytes, as indicated by the negative staining for Pref1, as well as a subcutaneous adipose layer, was present in the tissue sections of 36-week-old fetuses. In these sections, the expression of PRKAR2B was high (Fig. 1A), whereas that of PRKAR1A was nearly absent.

In 20- and 25-week-old fetuses, visceral adipose tissue exhibited intermediate features when compared with the tissue present during the first and the last few weeks of gestation, being composed of both preadipocytes (Pref1+) and morphologically distinct mature adipocytes (Pref1−). In the sections of retroperitoneal adipose tissue, the expression of PRKAR2B was high, whereas that of PRKAR1A was low (Fig. 1B), mimicking what was observed in adult human adipose tissue (12).

### PRKAR2B expression increases during adipogenesis in both murine and human cell models

**Figure 2:**
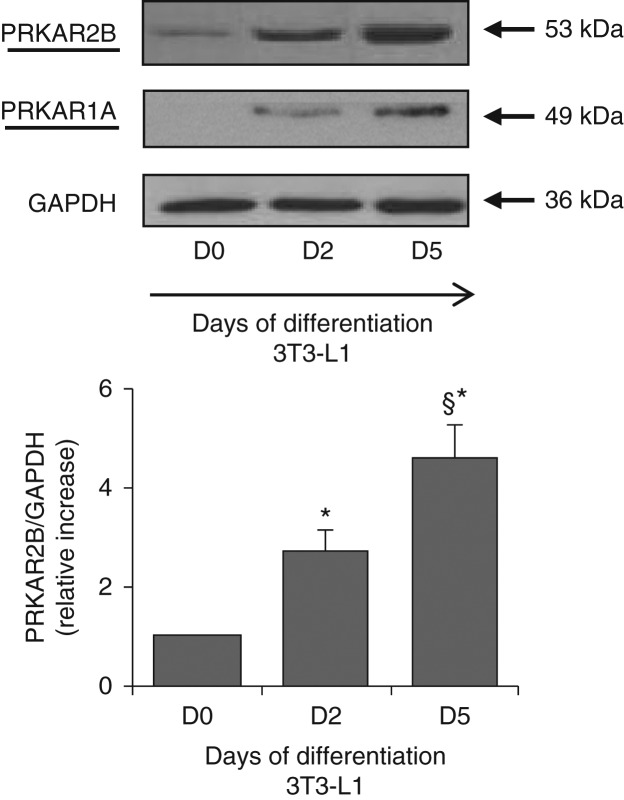
Representative western blot analysis of the expression of PRKAR2B and PRKAR1A proteins in 3T3-L1 cells carried out before (D0) and after 2 and 5 days of differentiation (D2 and D5) respectively. PRKAR2B was the most expressed PKA regulatory subunit at each stage of differentiation. The expression of PRKAR2B and PRKAR1A increased progressively until day 5 (D5) and then it remained constant. Data were normalized through GAPDH antibody hybridization. Chemiluminescence was detected using the ChemiDoc-IT Imaging System and analyzed using ImageJ. The values are expressed as relative increase when compared with the basal value, arbitrarily fixed as 1. All the experiments were repeated at least three times with similar results. Data are presented as means±s.d. *P<0.01 vs D0; ^§^*P*<0.05 vs D2.

PRKAR2B was the most expressed regulatory subunit in 3T3-L1 cells before and during *in vitro* differentiation. The expression of PRKAR2B was low in 3T3-L1 cells before differentiation, and it increased progressively from day 0 to day 5 of differentiation and then remained constant (Fig. 2). PRKAR1A was nearly absent before differentiation, and its expression increased progressively during the first few days of differentiation in 3T3-L1 cells, though remaining at lower levels when compared with that of PRKAR2B (Fig. 2).

**Figure 3:**
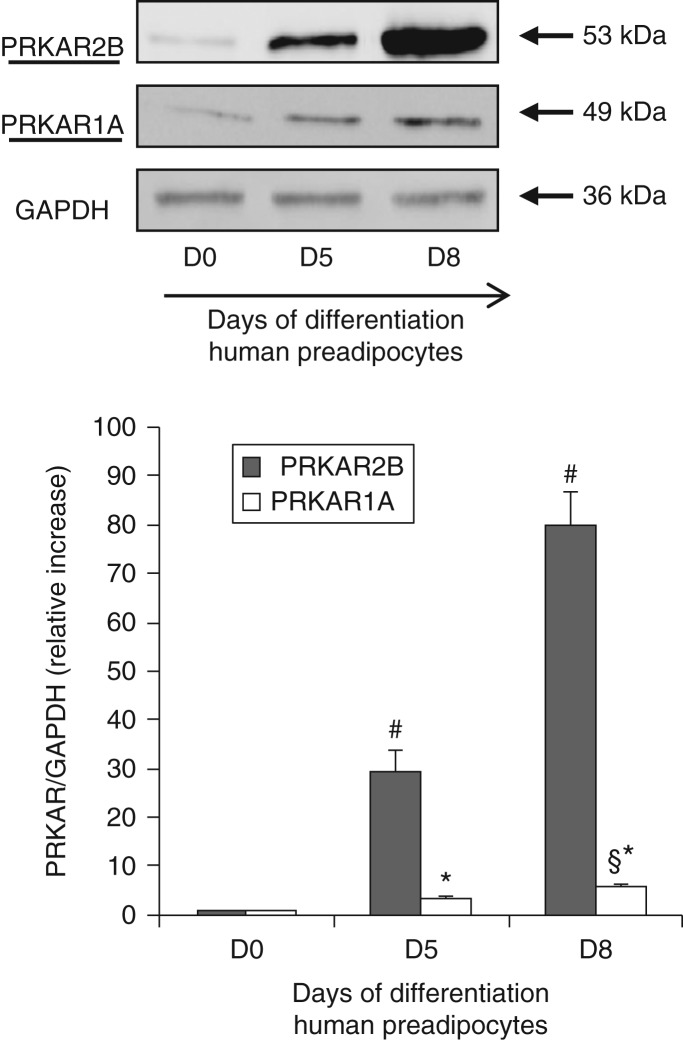
Representative western blot analysis of the expression of PRKAR2B and PRKAR1A proteins in primary human preadipocytes carried out before (D0) and after 5 and 8 days of differentiation (D5 and D8) respectively. PRKAR2B was the most expressed PRKA regulatory subunit at each stage of differentiation. The expression of PRKAR2B and PRKAR1A increased progressively until day 8 (D8) and then it remained constant. Data were normalized through GAPDH antibody hybridization. After digital acquisition, chemiluminescence was measured using ImageJ, and the values are expressed as relative increase when compared with the basal value, arbitrarily fixed as 1. At least three experiments with primary cultures from samples of different subjects were repeated with similar results. Data are represented as means±s.d. ^#^*P*<0.001 vs D0 and D5 respectively; *P<0.01 vs D0; ^§^*P*<0.05 vs D5.

PRKAR2B was also the most expressed regulatory subunit in primary human preadipocytes before and during differentiation, a pattern similar to that observed in 3T3-L1 cells. It is worth noting that the differentiation process was slower in human preadipocytes than in 3T3-L1 cells. In fact, the expression of PRKAR2B, which was low in undifferentiated preadipocytes, increased progressively after the start of differentiation and reached its highest levels after 8 days (Fig. 3) and remained constant thereafter. The expression of PRKAR1A was very low in primary human adipocytes before differentiation, and a slight increase was observed after 8 days of differentiation (Fig. 3).

### PRKAR2B is a positive regulator of adipogenesis

Since PRKAR2B was the most expressed regulatory subunit in our cell models and its expression increased progressively during adipocyte differentiation, we decided to investigate the effects of its silencing on adipogenesis. For this purpose, murine 3T3-L1 cells and primary human preadipocytes were transfected with siRNA against *PRKAR2B* to transiently reduce the expression of *PRKAR2B* during differentiation and thereafter induced by standard protocols.

**Figure 4:**
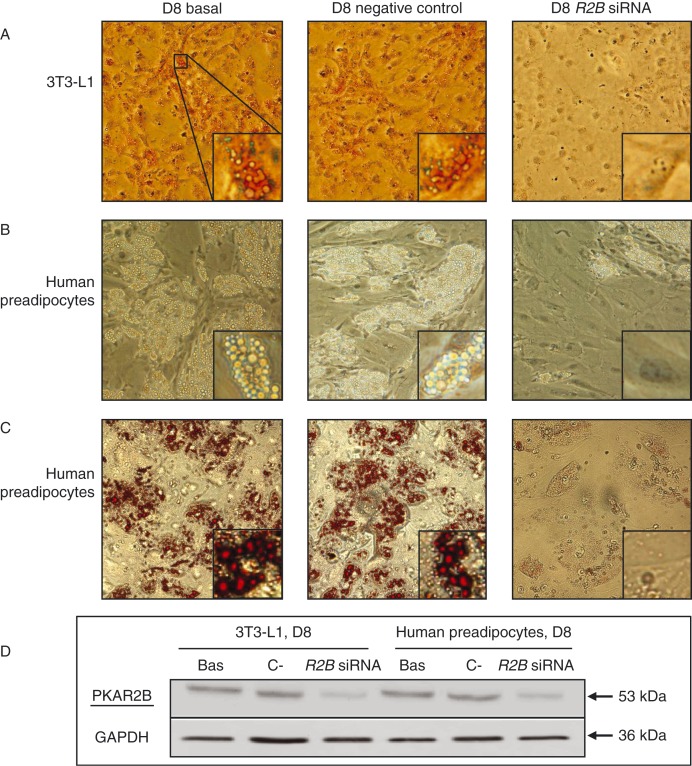
Representative images of intracellular lipid accumulation after differentiation of 3T3-L1 cells and primary human preadipocytes. siRNA against *PRKAR2B* inhibited 3T3-L1 and human preadipocyte differentiation. (A) 3T3-L1 cells. Oil Red O staining of nontransfected cells, cells transfected with the negative control siRNA, and cells transfected with siRNA against *PRKAR2B* respectively after 8 days of induction of differentiation. (B) Human preadipocytes. Phase-contrast images of nontransfected cells, cells transfected with the negative control siRNA, and cells transfected with siRNA against *PRKAR2B* respectively after 8 days of induction of differentiation. (C) Human preadipocytes. Oil Red O staining of nontransfected cells, cells transfected with the negative control siRNA, and cells transfected with siRNA against *PRKAR2B* respectively after 8 days of induction of differentiation. The cells were photographed using a phase-contrast microscope. Magnification ×20, inset: close-up view. (D) Immunoblots showing the expression of PRKAR2B in control cells and silenced cells on D8 of differentiation in both 3T3-L1 and human preadipocytes.

The progression of the physiological adipocyte differentiation process is characterized by a change in cell morphology from fibroblast-like preadipocytes to round-shaped cells. The increase in intracellular lipid accumulation, mainly as triglyceride droplets, can be visualized by Oil Red O staining. Using this approach, although lipid accumulation was found to be slower in human preadipocytes than in 3T3-L1 cells, triglyceride droplets were well visualized in both the cell types after 8 days, a time lag covered by the duration of transfection and silencing (Fig. 4D).

The silencing of *PRKAR2B* resulted in reduced triglyceride accumulation in both 3T3-L1 cells (Fig. 4A) and primary human preadipocytes (Fig. 4B and C), when compared with nonsilenced or negative control siRNA-transfected cells. As the increase in intracellular triglyceride accumulation observed after the application of adipogenic stimuli was similar in nontransfected cells and cells transfected with the negative control siRNA, a nonspecific effect of transfection on the inhibition of adipocyte differentiation was ruled out in both the cell systems (Fig. 4A, B, and C).

**Figure 5:**
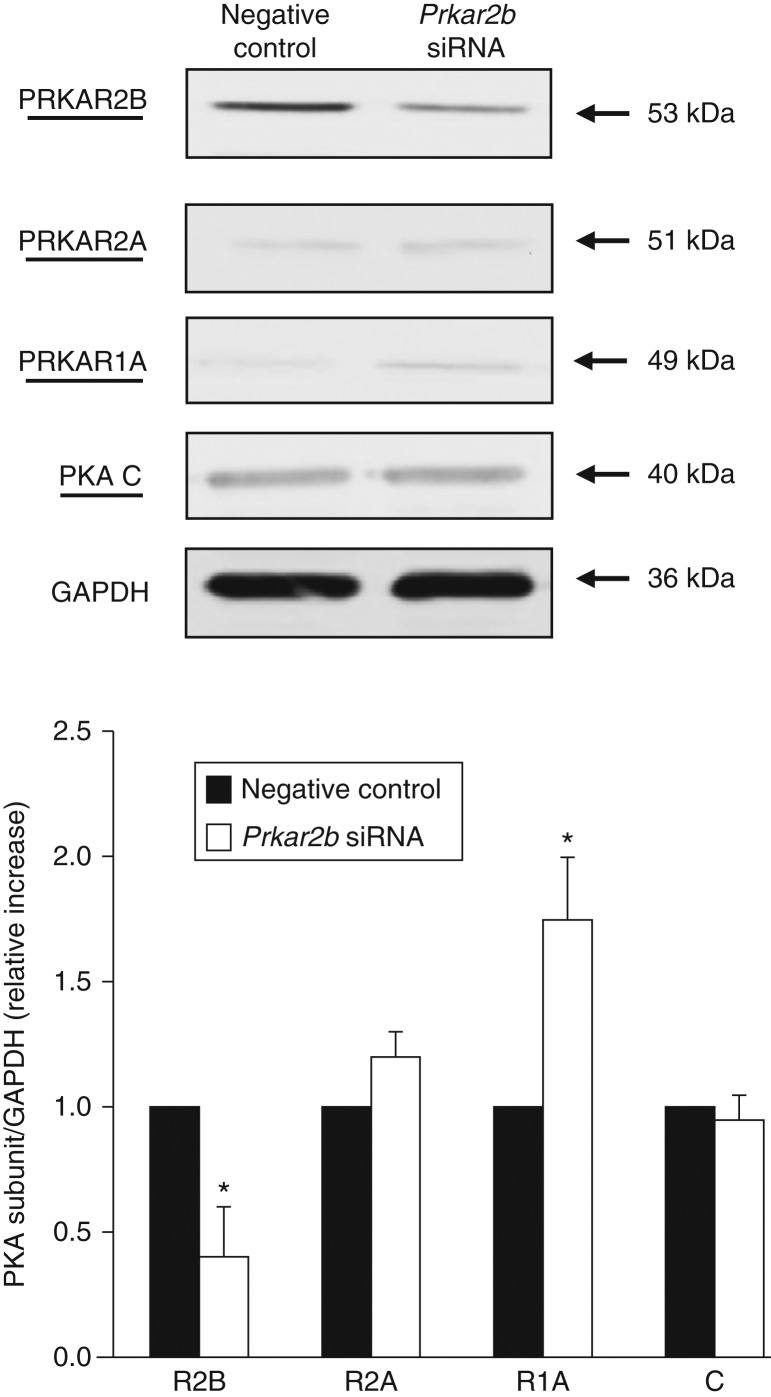
Effects of *R2B* silencing on the other subunits of PKA in 3T3-L1 cells. The cells were transfected for 72 h with negative control or *R2b* siRNA. Immunoblots with specific antibodies against PRKAR2B, PRKAR1A, PRKAR2A, and catalytic (C) subunits were obtained. Experiments were repeated three times with similar results, and a representative western blot is shown. Values are expressed as relative increase when compared with the basal value, arbitrarily fixed as 1. Data are presented as means±s.d. *P<0.01 vs basal value.

To investigate the effects of PRKAR2B ablation from the PKA complex, using western blot analysis, we evaluated the expression of other PKA subunits after 72 h of *PRKAR2B* silencing in 3T3-L1 and human preadipocytes (Fig. 5 and data not shown). Our data showed that in both the cell models, the expression of the PRKAR1A subunit was significantly increased after *R2B* silencing, suggesting a compensatory increase, as observed in other cell systems (13). By contrast, no differences in the expression of PRKAR2A, which was very low, and catalytic subunits were observed.

The exposure of 3T3-L1 cells to adipogenic stimuli initiates MCE, an event that precedes the expression of genes involved in adipocyte differentiation. However, it is still unknown whether this step is a prerequisite for differentiation. To determine whether the silencing of *PRKAR2B* might also have effects on the MCE process, we transfected cells with *R2B* siRNA or negative control siRNA, and we determined the number of cells 48 h after the induction of differentiation. Our results indicated an increase of about twofold in the number of cells induced to differentiate with respect to uninduced cells at the same time point. No differences were found between the control cells or *R2B*-silenced cells.

### *PRKAR2B* silencing abolished the increase in adipocyte differentiation molecular markers

**Figure 6:**
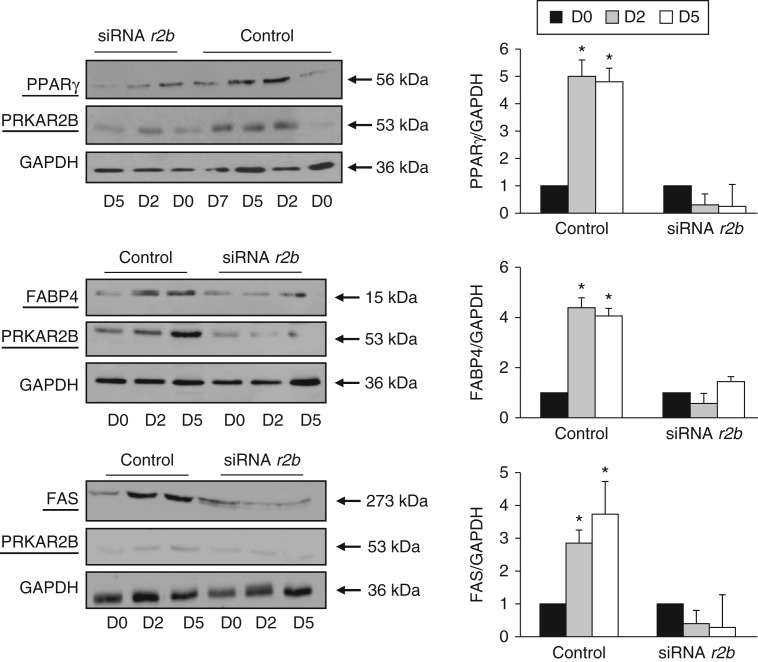
Representative western blot analysis of the expression of PPARγ, FABP4, and FAS in 3T3-L1 cells carried out before (D0) and after 2 and 5 days of differentiation (D2 and D5) respectively. In control cells transfected with the negative control siRNA, the expression of PPARγ, FABP4, and FAS increased progressively during the first few days of normal 3T3-L1 cell differentiation. In the *Pkar2b*-silenced cells, the expression of PPARγ, FABP4, and FAS did not increase after the administration of adipogenic stimuli. PPARγ antibody detected only an immunoreactive band at 56 kDa corresponding to PPARγ2 isoform. All the experiments were repeated at least three times with similar results. Values are expressed as relative increase when compared with the basal value, arbitrarily set as 1. Data are presented as means±s.d. *P<0.01 vs basal value.

**Figure 7:**
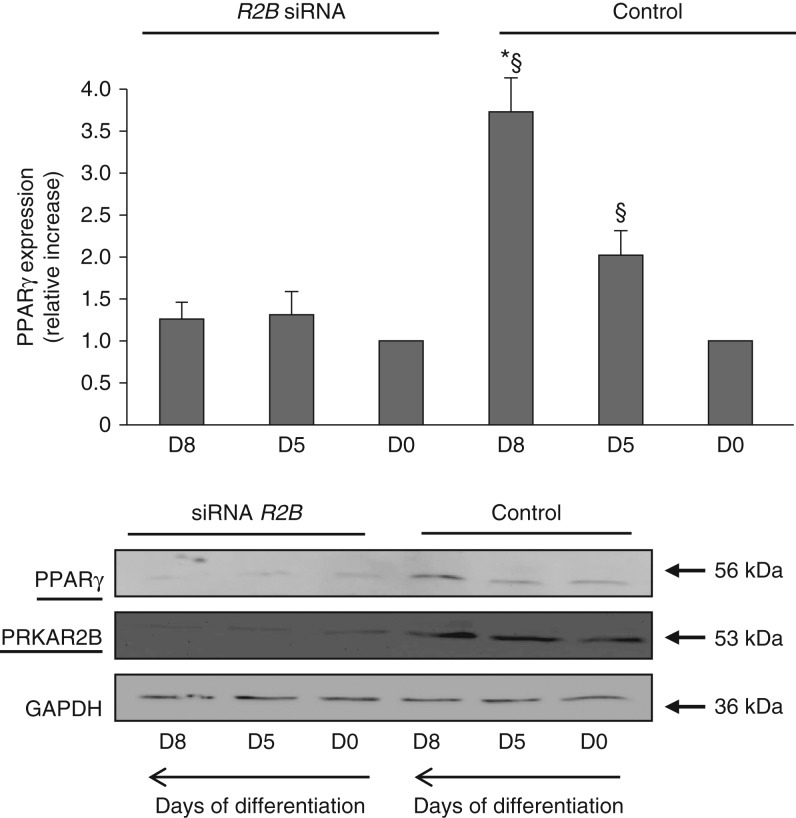
Representative western blot analysis of the expression of PPARγ in primary human preadipocytes carried out before (D0) and after 5 and 8 days of differentiation (D5 and D8) respectively. PPARγ antibody detected only an immunoreactive band corresponding to PPARγ2 isoform (56 kDa). The expression of PPARγ increased progressively during adipocyte differentiation (negative control cells). The silencing of *PRKAR2B* was effective before and during the differentiation of primary human preadipocytes. The expression of PPARγ did not increase after the administration of adipogenic stimuli in human preadipocytes transfected with siRNA against *PRKAR2B*. Data were normalized through GAPDH antibody hybridization. Values are expressed as relative increase when compared with the basal value, arbitrarily fixed as 1. All the experiments were repeated at least three times with similar results. Data are presented as means±s.d. ^§^*P*<0.05 vs D0 and D5 respectively; *P<0.01 vs D0.

During the first few days of differentiation, both 3T3-L1 and primary human preadipocytes transfected with the negative control siRNA exhibited a progressive increase in the expression of PPARγ, with the highest expression occurring on days 5 and 8 respectively and remaining constant thereafter (Figs 6 and 7, control cells). The silencing of *Prkar2b* abolished the increase in the expression of PRKAR2B during differentiation. Most importantly, under these experimental conditions, the expression of PPARγ did not increase in 3T3-L1 and primary human preadipocytes, when compared with control cells.

Accordingly, the silencing of *PRKAR2B* in 3T3-L1 cells also completely abolished the increase in the expression of FABP4 and FAS during the induction of differentiation (Fig. 6).

**Figure 8:**
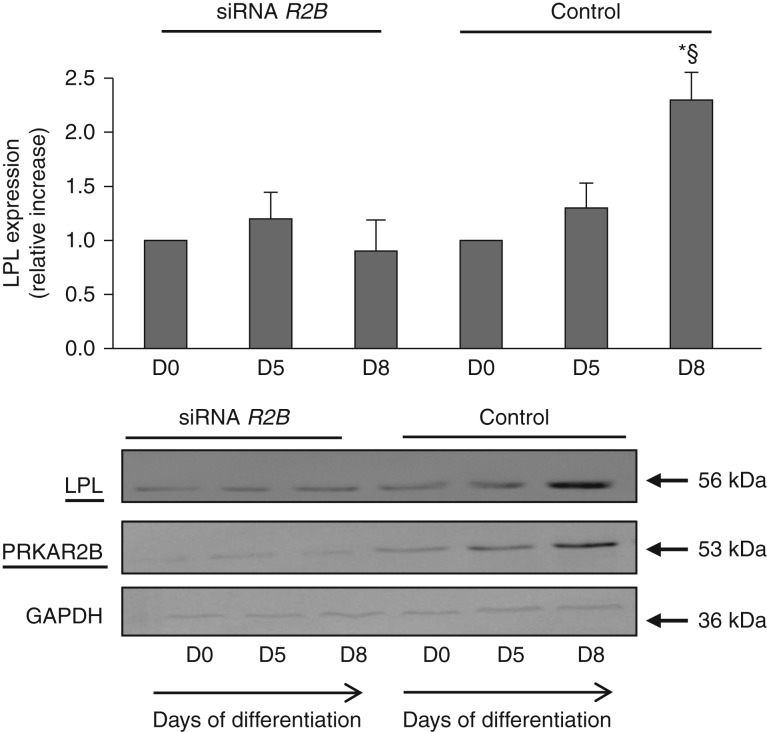
Representative western blot analysis of the expression of LPL in primary human preadipocytes carried out before (D0) and after 5 and 8 days of differentiation (D5 and D8) respectively. The expression of LPL increased during the differentiation of human adipocytes (negative control cells). The expression of LPL did not increase after the administration of adipogenic stimuli in human preadipocytes transfected with siRNA for *PRKAR2B*. Data were normalized through GAPDH antibody hybridization. The values are expressed as relative increase when compared with the basal value, arbitrarily fixed as 1. All the experiments were repeated at least three times with similar results. Data are presented as means±s.d. *P< 0.01 vs D0; ^§^*P*<0.05 vs D5.

Moreover, the expression of LPL, a marker of complete adipocyte differentiation, was also impaired in silenced primary human preadipocytes, when compared with control cells (Fig. 8).

## Discussion

In this study, we evaluated the role of PKA regulatory subunits in adipogenesis, as an extension of previous investigations carried out in our laboratory (12). Our study first demonstrated that PRKAR2B is highly expressed in human fetal tissues and is required for adipocyte differentiation *in vitro*.

In line with previous reports on mice and adult humans (11, 12, 13), the present study provides data indicating that PRKAR2B is the key PKA regulatory subunit in human adipose tissue during fetal development. Little is known about the formation of WAT during human development, and most of the data concerning adipocyte differentiation have been obtained from *in vitro* studies (1, 2). Interestingly, while in rodents WAT cannot be macroscopically detected during embryogenesis and develops mainly after birth, in human fetuses the formation of WAT starts well before birth (18). In accordance with the study of Poissonnet *et al*. (19), we detected preadipocytes in periorbital tissue at 12 weeks of gestation using the preadipocyte marker Pref1, an epidermal growth factor-like domain-containing transmembrane protein expressed by adipocyte precursors and known to inhibit adipocyte differentiation (20, 21). Our data indicated that during the early phases of fetal development PRKAR2B is the only PKA regulatory subunit expressed in human adipose tissue where it co-localizes with Pref1. In the subsequent phases and until the end of gestation, when fetal adipose tissue has nearly acquired the features of adult adipose tissue, the expression of PRKAR2B is high, while Pref1 disappears, due to the decrease in the amount of preadipocyte precursors.

It is well established that there are distinct and cell-specific roles for the different PKA isoenzymes in the regulation of growth control and differentiation (7, 10, 17, 22, 23, 24, 25). In particular, studies in *Prkar2b* knockout mice have shown that these animals remain remarkably lean due to increased metabolic activity (13), probably due to compensatory increase in the expression of PRKAR1A regulatory subunit. However, *PRKAR2B* mRNA levels have been found to be significantly lower in adipose tissue of obese patients than in that of nonobese patients and to be negatively correlated with BMI, waist circumference, and insulin levels, consistent with the hypothesis that the expression and activation of PRKAR2B might contribute to the different lipolytic response to β-adrenergic activation in obesity (12). In the light of the results of the present study, demonstrating the effects of *PRKAR2B* silencing in cultured cells, it appears that further studies are needed to investigate the role of PRKAR2B in adipogenesis *in vivo* and to determine whether a decrease in lipolysis would be sufficient to explain obesity and the possible relative involvement of adipogenesis.

This is the first study, to our knowledge, to investigate the expression and role of PKA regulatory subunits in an experimental cell model of adipogenesis. In this model, we found that PRKAR2B was the key regulatory subunit in both murine and human preadipocytes during adipogenesis. The increase in the expression of PRKAR2B during the first few days of adipocyte differentiation *in vitro* might *per se* suggest a role for this regulatory protein in the progression of adipogenesis. This hypothesis was confirmed by transient transfection with siRNA against *PRKAR2B* of both immortalized 3T3-L1 murine cells, one of the most frequently used preadipocyte lines, and primary human preadipocytes, which better reflects adipose tissue biology *in vivo*. Data obtained from both the experimental models showed that the silencing of *PRKAR2B* resulted in the expected abolishment of the increase in the expression of PRKAR2B during adipogenesis that was associated with an impaired lipid accumulation in differentiating adipocytes.

Adipogenesis involves a cascade of transcription factors, among which *PPARγ* and *C/EBP*s are considered to be the crucial determinants of adipocyte fate. However, despite the importance of *C/EBP*s in adipogenesis, these transcription factors cannot function efficiently in the absence of *PPARγ*. *PPARγ*, the master regulator of adipogenesis, belongs to the nuclear receptor superfamily of ligand-activated transcription factors and is both necessary and sufficient for adipogenesis (26). In fact, most pro-adipogenic factors seem to function, at least in part, by activating the expression and/or activity of PPARγ and, to date, no factor that can rescue adipogenesis in the absence of PPARγ has been identified. The endogenous PPARγ ligand is unknown; however, it has recently been demonstrated that increases in cAMP levels result in the transient production of a biologically relevant PPARγ ligand in 3T3-L1 cells during adipogenesis (27). Moreover, the activation of CREB promotes the expression of PPARγ (6). In the present study, we found that the expression of PPARγ did not increase in the absence of the physiological rise in the expression of PRKAR2B during adipocyte differentiation in both murine and human cell models. In turn, this event led to the abolishment of lipid accumulation in adipocyte progenitors, although stimulated to differentiate. Moreover, the expression of LPL, a protein associated with terminal differentiation of adipocytes, was also impaired by blocking the expression of *PRKAR2B*. This observation is consistent with results from previous studies demonstrating that embryonic stem cells lacking PPARγ do not contribute to fat formation (28, 29) and that the abolishment of the expression of PPARγ at the adipose tissue level prevents WAT development (30, 31, 32). In accordance with this, humans with dominant-negative *PPARγ* mutations display abnormal body fat distribution, demonstrating a crucial role for PPARγ also in human adipose tissue development (33, 34). In the models used in the present study, in which the expression of PPARγ was not selectively abolished, we found that the silencing of *PRKAR2B* significantly interfered with the normal expression of PPARγ and LPL. Although the pathway by which intracellular cAMP may influence the expression of PPARγ is still unclear, we are tempted to speculate that PKA holoenzyme may be involved in cAMP-mediated PPARγ induction.

In conclusion, the present study shows that PRKAR2B is the key PKA regulatory subunit in mouse and human adipose tissue development. PRKAR2B is the main regulatory subunit expressed in human adipose tissue during fetal development and the increase in its expression during murine and human adipocyte differentiation seems to be an essential event in adipogenesis.
